# Genetic susceptibility to burnout in a Swedish twin cohort

**DOI:** 10.1007/s10654-012-9661-2

**Published:** 2012-03-03

**Authors:** Victoria Blom, Gunnar Bergström, Lennart Hallsten, Lennart Bodin, Pia Svedberg

**Affiliations:** 1 Division of Insurance Medicine, Department of Clinical Neuroscience, Karolinska Institutet, Stockholm, Sweden; 2 Division of Intervention and Implementation Research, The Institute of Environmental Medicine, Karolinska Institutet, Stockholm, Sweden

**Keywords:** Burnout, Gender, Heredity, Twin study

## Abstract

Most previous studies of burnout have focused on work environmental stressors, while familial factors so far mainly have been overlooked. The aim of the study was to estimate the relative importance of genetic influences on burnout (measured with Pines Burnout Measure) in a sample of monozygotic (MZ) and dizygotic (DZ) Swedish twins. The study sample consisted of 20,286 individuals, born 1959–1986 from the Swedish twin registry who participated in the cross-sectional study of twin adults: genes and environment. Probandwise concordance rates (the risk for one twin to be affected given that his/her twin partner is affected by burnout) and within pair correlations were calculated for MZ and DZ same—and opposite sexed twin pairs. Heritability coefficients i.e. the proportion of the total variance attributable to genetic factors were calculated using standard biometrical model fitting procedures. The results showed that genetic factors explained 33% of the individual differences in burnout symptoms in women and men. Environmental factors explained a substantial part of the variation as well and are thus important to address in rehabilitation and prevention efforts to combat burnout.

## Introduction

Burnout is a stress-related phenomenon that has received widespread attention as an important problem for the society as well as for the affected individuals, and a large body of scientific publications has treated this subject from various views. Burnout has been found to be prospectively associated with a number of important negative outcomes such as poor job-performance [1], psychological ill-health [2], physical ill-health [3], self-reported sickness absence [4], long-term sickness absence [5], intent to leave the profession [6], suicidal ideation [7], and all-cause mortality [8]. Although many contributing factors to burnout have been studied, more knowledge of the underlying causes of burnout is needed, especially of the degree to which burnout is influenced by genetic and early environmental factors. So far, these issues have barely been examined at all [9, 10].

Presumably, burnout has an essential part of its origin in the broad economic and social changes, such as growing competition, individualism and work reorganizations that have taken place in modern societies [11]. The psychological pressure on people resulting from these changes has often found its expressions in feelings of frustration, depleted energy, lowered motivation, and identity threats. Such experiences have advantageously been captured by the construct of burnout. Initially, it was thought that burnout only occur among those persons working in the human services [12]. However, studies from the last decades have shown that burnout can be observed in almost any type of occupational group [13] but also among university students [14], and athletes [15]. The most frequently used measurement of burnout, the Maslach burnout inventory (MBI) [16] is restricted to the working population, while another commonly applied instrument, Pines Burnout Measure (Pines BM) [17] is a context free measure which is applicable and measures symptoms of burnout, in any group, such as students, unemployed people and people on sick-leave. The composite score of Pines BM correlates substantially with the exhaustion dimension of MBI [18], which has been held as the central aspect of burnout [19]. MBI and Pines BM have been found to equally well distinguish between burned out and non-burned out individuals [20].

Previous research indicates that a variety of job characteristics such as work load, role conflict, lack of social support at work, and little participation in decision making are of importance for burnout [21]. In addition to such contextual factors, demographic variables such as gender and age have been found to be related to burnout. According to two meta-analyses [22, 23], young people and women tend to be slightly more exhausted than older people and men. In addition, personality variables, which have clear genetic components [24] have demonstrated consistent links to burnout [25, 26]. These associations suggest that burnout may have a certain genetic origin.

A powerful tool in research of familial influences (genetic and shared/early environmental) of burnout would be to use a genetically informative population, such as a twin setting. Differences in similarity between identical (monozygotic) and fraternal (dizygotic) twins provide information about both genetic and environmental effects. Twin studies make use of the fact that monozygotic (MZ) twin pairs share all of their genes whereas dizygotic (DZ) twin pairs share on average half of their segregating genes. Consequently, if MZ and DZ twin pairs show the same degree of similarity, environmental factors are most important for the trait studied, while higher concordances among MZ than DZ twin pairs indicate that genetic factors also are of importance for the trait under study. To our knowledge, only two twin-family studies on the topic have been presented to date [9, 10]. These studies showed a familial clustering of burnout, and that the clustering was due to genetic factors in men, while for women both genetic and shared environmental factors were of importance. Inclusion of opposite-sexed (OS) twins is essential for evaluating whether different sets of genes or different shared environments are operating in the two sexes. To our knowledge, there are no reports that include opposite-sex twins and specifically test whether different genes are of importance for individual differences in burnout symptoms in the two sexes.

The aim of the present study was to estimate the relative importance of genetic and environmental influences on burnout using Pines BM in a population based Swedish twin cohort including same- and opposite sexed twins.

## Methods

### Participants

The source population consisted of twins from the Swedish twin registry (STR) born 1959–1985 and who participated in the STAGE (study of twin adults: genes and environment) web-based questionnaire in 2005 [27]. In total 25,378 twin individuals, whereof 56% were women, answered the questionnaire. The source population represents various groups such as students (6%), unemployed (3%), individuals on maternity leave (4%) and individuals on sick leave or disability pensioned (2%) and workers in various professions and sectors (47% full time employed). Excluded from the analyses were twins with unknown zygosity and individuals due to non-response to any of the three items included in the Pines BM (*n* = 5,092).

Hence, a total of 20,286 individuals with complete information on Pines BM and zygosity were included in the analyses. Of these were 7,110 complete twin pairs, 5,103 were same sexed (3,038 MZ and 2,065 DZ) and 2,007 were OS twin pairs. Missing data analysis showed that non-respondents were comparable to the respondents as regards zygosity (34% MZ, 29% same sex DZ and 38% OS DZ compared to 41% MZ, 29% same sex DZ and 30% OS DZ in the study group), as well as for burnout for twin partner to non-respondents (M = 2.51, SD = 1.28 compared to M = 2.57, SD = 1.33 in the study group). Mean values and standard deviation values of burnout on item level were equivalent among the respondents and non-respondents. The gender balance was more equal in the missing data (49% men and 51% women compared to 38% men and 62% women in the study group).

Zygosity determination for like-sexed twin pairs was obtained in the STAGE-study on the basis of questions about childhood resemblance. When validated against serological and micro-satellite markers this method is about 98% accurate [28].

### Measures

Burnout was measured with three items from the Pines BM [17], expressed as the adjectives “feeling depressed”, “being emotionally exhausted” and “feeling run down”. Answers were given by respondents on a seven point Likert scale ranging from “1 = do not agree” to “7 = agree entirely”. In line with other studies analyzing burnout [e.g. 20, 29] item responses were summed and divided by the number of items (i.e. 3) in order to get the mean value of burnout for each individual, ranging between 1 and 7. A high score indicates higher burnout level. Burnout was treated as a continuous variable, since Pines BM concerns symptoms rather than pathology, but also as a dichotomous variable. In the dichotomous variable, the cut off limit for burnout versus no burnout was set to 4.0 in accordance with a Swedish population study of burnout [30]. Further, the three items of Pines BM included in STAGE and hence available for the present study, were chosen as they were found to correlate strongly (*r* = 0.90) with the full 21 item Pines BM [29]. In the present study, Cronbach’s α for the three-item scale was 0.89.

### Statistical analyses

Within pair correlations (Pearson) were calculated with burnout as a continuous variable. Probandwise concordance rates (the risk for one twin to be affected given that his/her twin partner is affected) and tetrachoric correlations (*r*) were calculated for same-sexed MZ, and same—and opposite sexed DZ twin pairs on the dichotomous burnout variable. The prevalence of burnout in each zygosity group by sex was calculated as the number of individuals with burnout based on the dichotomous variable compared to all individuals in the group. The risk of burnout was estimated as an odds ratio (OR) with 95% confidence intervals (CI) where the odds for participants with burnout having a twin partner with burnout was compared to the odds for burnout in a twin whose co-twin did not have burnout.

The relative importance of genetic and environmental factors for burnout were calculated with standard biometrical model fitting procedures with raw data (continuous Pines BM variable) using Mx [31]. The aim of quantitative genetic analysis is to determine the extent to which genetic and environmental influences are important for variation in a trait, in this case burnout. MZ twins are genetically identical, whereas DZ twins share, on average, 50% of their segregating genes. Two sources of genetic influence can be estimated: additive genetic variation, which is the sum of the effects of all alleles affecting the phenotype, and dominance, the part of the genetic variation due to interaction between alleles at the same locus. Epistatic genetic effect, i.e. interaction of alleles between different loci, is assumed to be absent. Additive and dominance genetic effects have a correlation of 1.0 within MZ pairs and 0.5 and 0.25 within DZ pairs, respectively [32]. Hence, if additive genetic influences (A) or dominant genetic influences (D) are important for burnout, then MZ twins should be significantly more similar than DZ twins. Shared environmental influences (C) refer to non-genetic influences that contribute to similarity within pairs of twins regardless of zygosity, such as shared family environment, uterine environment and contact throughout life. Nonshared environmental influences (E) are those individual specific influences (e.g. accidents, illnesses, different life experiences or occupations) that make family members different from one another, including measurement error. Structural Equation Modeling is commonly employed to provide maximum-likelihood estimates of percents of total variance (*a*², *c*²/*d*² and *e*²). The significance of parameters was evaluated through nested model comparisons. The fit of the nested models was analysed by log-likelihood tests. The difference in the −2 LL values and corresponding degrees of freedom is distributed as a χ^2^. If the difference in the log-likelihoods between two nested models associated with the difference in degrees of freedom ($\Updelta \chi^{ 2}_{df}$) is statistically significant, the more parsimonious model fits significantly worse and lacks important parameters. Akaike’s information criterion (AIC), reflecting both the goodness of fit of the model and its parsimony, was computed and the model with the lowest (i.e. largest negative) AIC value is said to fit best. Studies of like-sexed twins enable one to evaluate whether there are sex differences in the total variance and whether there are sex differences in the relative importance of genetic and environmental influences. Including OS pairs provides an opportunity to test whether *different* genes and *different* shared environments are operating in the two sexes [32]. Lower within pair correlations for the opposite sexed twins than for the like-sexed DZ twins suggest a sex-specific effect, i.e. that different genes or shared environments are operating in men and women. In order to obtain parameter estimates for *a*², *c*²/*d*² and *e*² and parameters, r_a_ and/or r_d_, which indicates whether genetic effects are the same or different in males and females, we used all six twin groups (MZ female, MZ male, DZ female, DZ male, OS male–female, OS female–male) simultaneously and a series of models was tested. The heritability (*h* ^2^) coefficient refers to the proportion of the total variation in the trait, here burnout symptoms, which is due to genetic difference between individuals [33, 34]. The coefficient ranges between 0 and 1.0 and a coefficient over 0.50 is considered substantial [35].

Decisions about fitting ACE models versus ADE models were made based on the pattern of the within pair correlations and by comparing the AIC values of the ACE and the ADE model.

The study was approved by the Regional Ethical Review Board in Stockholm, Sweden.

## Results

The level of burnout measured with Pines BM differed significantly between men (M = 2.21) and women (M = 2.79) (*F* = 487, 94; *P* < 0.001) (Table 1). Results based on the dichotomization of Pines BM are presented in Table 2. The prevalence of burnout based on the binary variable was 11.8% for men and 25.9% for women. As a consequence, the number of pairs where both twins were classified as being burned out (concordant affected) differed largely between men and women. There were only six DZ male twin pairs, compared to 110 DZ female twin pairs, concordant for burnout. All estimates showed a difference between men and women. The probandwise concordance rates and tetrachoric correlations were markedly higher for MZ than for DZ twins, for both men and women. A similar pattern of higher estimates for MZ than DZ twin pairs was shown for Pearson correlation coefficients calculated using Pines BM as a continuous variable measuring burnout symptoms (Table 1). Somewhat lower within pair correlations for the opposite sexed twins than for the like-sexed DZ twins suggest a sex-specific effect, i.e. that different genes or shared environments are operating in men and women. An assumption of quantitative genetic analyses based on twin data is that variances are equal for MZ and DZ twins. Analyses of variance indicated that there were no differences in means and variances between MZ and DZ twins for burnout. Further, the ORs were statistically significant for all zygosity groups by sex except for DZ men. ORs were higher for MZ than DZ twins and the 95% CI did not overlap between MZ and DZ women, or between MZ and DZ men (Table 2).

**Table 1:** Number of twin pairs (*n*), mean values and standard deviations (SD), and within pair correlations (Pearson correlation) for Pines Burnout Measure (continuous variable) by zygosity and sex

Zygosity	Sex	*n*	Mean	SD	Within pair correlation
MZ	Women	1,887	2.82	1.38	0.33
	Men	1,151	2.22	1.19	0.34
	Total	3,038	2.59	1.34	0.36
DZ	Women	1,282	2.75	1.38	0.14
	Men	783	2.18	1.13	0.07
	Total	2,065	2.54	1.32	0.16
OS		2,007	2.49	1.30	0.09

*MZ* monozygotic, *DZ* dizygotic, *OS* opposite sexed DZ

**Table 2:** Prevalence, probandwise concordance rates, tetrachoric intra pair correlations, and OR with 95% CI for Pines Burnout (Pines BM) Measure (binary variable) in a Swedish cohort of MZ, same-sexed DZ and opposite sexed (OS) twins

Participants	Concordant pairs Pines BM (*n*)	Discordant pairs (*n*)	Concordant pairs no Pines BM (*n*)	Concordance rates	Tetrachoric correlation	OR (95% CI)	Prevalence Pines BM (%)
All							
MZ	268	756	2,014	0.42	0.44	3.78 (3.30–4.33)	19.7
DZ	116	587	1,362	0.28	0.20	1.83 (1.54–2.19)	18.1
OS	92	587	1,328	0.24	0.11	1.42 (1.18–1.71)	16.9
Men							
MZ	48	201	902	0.32	0.44	4.29 (3.23–5.69)	12.0
DZ	6	156	621	0.07	0.13	0.61 (0.33–1.13)	10.7
Women							
MZ	220	555	1,112	0.44	0.40	3.18 (2.72–3.71)	26.4
DZ	110	431	741	0.34	0.20	1.76 (1.45–2.13)	25.4

As the comparison of the AIC values of the ACE and the ADE univariate models was in favour of the ADE model (even though differences seem to be minor), a sex-limitation ADE model for Pines BM continuous scores was applied to find the best fitting and most parsimonious model. Detailed model fit statistics are presented in Table 3. In a sex-limitation model using all zygosity groups, the sex-specific genetic effects were found to be statistically non-significant, indicating that the same genes were accounting for genetic effects in burnout in both men and women (*h* ^2^ = 33%), however the relative magnitude (A and D) nonetheless differed somewhat between the sexes (Table 4). The remaining variance (67%) was explained by non-shared environmental variance. Proportions of trait variance explained by genetic and environmental factors including 95% CI are shown in Table 5.

**Table 3:** Model fit statistics for univariate ACE and ADE models for Pines Burnout Measure in a cohort of Swedish twins

Model	−2 × log-likelihood	*df*	∆*df*	∆χ^2^	*P* value	AIC
ACE	66,673.356	20,195				26,283.356
CE	66,758.113	20,197	2	84.757	0.000	26,364.113
ADE	66,668.294	20,194				26,280.294
AE	66,673.356	20,196	2	5.062	0.080	26,281.356
E	67,090.598	20,198	4	422.304	0.000	26,694.598

*df* degrees of freedom, *AIC* Akaike’s information criterion

Phenotypic variation decomposed into additive (A) and dominance (D) genetic variation, shared (C) and unique (E) environmental variation

**Table 4:** Model fit statistics for sex-limitation ADE model of Pines Burnout Measure

Model	−2 × Log-likelihood	*df*	∆*df*	∆χ^2^	*P* value	AIC
ADE men ≠ women, r_a_/r_d_ free	66,668.29	20,194				26,280.29
ADE men = women, r_a_/r_d_ free	66,884.36	20,197	3	216.07	0.00	26,490.36
ADE men = women, r_a_/r_d_ fixed (0.5, 0.25)	66,884.36	20,199	5	216.07	0.00	26,486.36
ADE^a^ men ≠ women, r_a_/r_d_ fixed	66,668.29	20,196	2	0.00	1.00	26,276.29
AE men ≠ women, r_a_/r_d_ free	66,673.35	20,196	2	5.06	0.08	26,281.36
AE men ≠ women, r_a_ fixed	66,676.04	20,198	4	7.74	0.10	26,280.04
AE men = women, r_a_/r_d_ free	66,887.92	20,198	4	219.62	0.00	26,491.92
AE men = women, r_a_ fixed	66,890.03	20,200	6	221.74	0.00	26,490.03
E men ≠ women	67,090.59	20,198	4	422.30	0.00	26,694.60

^a^Best model: ADE males ≠ ADE females, but DOS A(D)-correlation (r_a_ and r_d_) = DZ A(D)-correlation i.e. quantitative differences but no qualitative differences (same genes, different amounts of A/D)

**Table 5:** Proportion of trait (Pines Burnout Measure) variance explained by genetic (A and D) and environmental (E) factors, with 95% CI

Sex	*a* ^2^	*d* ^2^	*e* ^2^
Men	0.10 (CI 0.01–0.29)	0.23 (CI 0.02–0.34)	0.67 (CI 0.62–0.72)
Women	0.23 (CI 0.04–0.35)	0.11 (CI 0.00–0.30)	0.67 (CI 0.63–0.71)

*a* ^2^ Proportion of trait variation explained by additive genetic factors, *d* ^2^ proportion of trait variation explained by dominance genetic factors, *e* ^2^ proportion of trait variation explained by non-shared environmental factors

## Discussion

The aim of the study was to estimate the relative importance of genetic influences on burnout measured with Pines BM. The results show a genetic susceptibility to burnout. The heritability was 33% and equal for women and men. One previous study [10] showed that burnout, as measured with the exhaustion dimension in MBI, clustered in families, but that this primarily was due to the environment shared by family members rather than by genetic factors as the difference between MZ and DZ twin pairs was not significant in their study. However, a more recent study by Middeldorp et al. [9] showed that the familial clustering was due to genetic factors in men, and genetic and shared environmental factors in women. The same study showed that for the exhaustion dimension in the MBI, heritability was about 30% for men and 13% for women. This coincides partly with the results in the present study, with a genetic effect of 33%, which is similar to the heritability for men in the previous study. The lack of evidence for shared environmental effect in the present study suggests only that its influence is less powerful than the dominant genetic influences, not that shared environmental effect would be non-existent for burnout.

It is also important to note that heritability is dependent upon the environment, as it refers to a specific population at a certain point in time [36]. One misinterpretations of the heritability coefficient is that it provides an index of trait malleability, i.e. the higher the heritability the less modifiable the trait is through environmental intervention [33]. This is not the case since the heritability coefficient refers to the proportion of the total variation in the trait which is due to genetic difference between individuals. Even though the results of the present study show that genetic factors play a role in burnout symptoms at the population level, environmental factors also explain a substantial part of the variation, i.e. 67%. This is important knowledge for practitioners working with rehabilitation and prevention of burnout as this means that also stressors in the work environment and in the private life are likely to be important in the aetiology of burnout, as suggested by other researchers [21, 37, 38]. Further, it is also possible that a variety of different environmental factors interact in burnout symptoms such as double work burden in paid as well as in unpaid household work [39].

The present results indicate an equal heritability of burnout for women and men, but possibly including some genetic differences as shown by different proportions of variance explained by additive and dominant effects for women and men. However, further research is needed to explore this finding. In the present study the difference between models (ACE and ADE) was minor, and the models constraining the genetic (additive and dominant) correlations of OS twin pairs to be equal to DZ same sexed pairs fit best. Similar results of presence of dominance influences have been demonstrated for self-esteem [40] and other personality variables [33], i.e. traits that potentially are related to burnout.

There are limitations as well as strengths in the present study. There was some internal missing data in Pines BM. However, missing data analysis showed the drop outs being representative for the study group. Also, results from a previous study that investigated whether non-response reduced the effective sample size, and hence might introduce bias in twin studies, showed a non significant finding of differences in burnout scores for the individuals in incomplete twin pairs compared to individuals from complete twin pairs [41]. Limitations also include the assumptions of twin analysis such as random mating and equal environments. Previous twin studies suggest that random mating is present for personality traits [42], and that shared environmental correlations between MZ and DZ twins are the same [43], For burnout the presence of random mating is not known but it could be supposed that burnout is more comparable to personality than to for example height, where assortative mating is found to be substantial [44]. A strength is that the present study is based on data from the large population based STR. However, future studies could benefit from better balance between men and women and the zygosity groups. The present study used Pines BM instead of the most frequently used scale MBI, which could complicate comparisons between studies, although Pines BM has been found to be strongly associated with the exhaustion dimension of MBI [18]. An advantage with Pines BM, as being a context-free burnout scale, is that groups outside the labor market, such as students, job-seekers and home-workers also can participate in studies of burnout. This is relevant as recent studies have shown that most occupational groups and various groups outside of labor market, such as students, can be affected by burnout symptoms [14]. Finally, the prevalence in the present study (25.9% for women and 11.8% for men) is considered to be high as compared to other studies [29]. Very high levels of burnout have however been noted [7] and the high prevalence could result from the participants being fairly young (19–47 years old) and with a majority of female participants, as some studies show that young age and female sex is associated with burnout [22, 23].

## Conclusions

In sum, the present study has shown that genetic factors are of importance for individual differences in burnout for both women and men. Although genetic factors play an important role, environmental factors explain a large part of the variation in burnout as well, and are thus important to address in rehabilitation and prevention efforts to combat burnout. However, studies focusing on the impact of stress on burnout could benefit from considering familial factors as confounders.
